# Correction to: miR-3928v is induced by HBx via NF-κB/EGR1 and contributes to hepatocellular carcinoma malignancy by down-regulating VDAC3

**DOI:** 10.1186/s13046-020-01655-2

**Published:** 2020-08-21

**Authors:** Qiaoge Zhang, Ge Song, Lili Yao, Yankun Liu, Min Liu, Shengping Li, Hua Tang

**Affiliations:** 1 Tianjin Life Science Research Center and Department of Pathogen Biology, Collaborative Innovation Center of Tianjin for Medical Epigenetics, School of Basic Medical Sciences, Tianjin Medical University, No. 22 Qi-Xiang-Tai Road, Tianjin, 300070 China; 2 The Cancer Institute, Tangshan People’s Hospital, Tangshan, 063001 China; 3 Department of Hepatobiliary Oncology, State Key Laboratory of Oncology in Southern China, Cancer Center, Sun Yat-sen University, Guangzhou, 510060 China

**Correction to: J Exp Clin Cancer Res 37, 14 (2018)**

**https://doi.org/10.1186/s13046-018-0681-y**

Following publication of the original article [1], the authors identified some errors in Figs. 4, 5 and 6; specifically panels Fig. 4d, Fig. 5e, and Fig. 6h. The corrections do not change the results and conclusions of this paper.

The correct figures are given below.

**Fig. 4:**
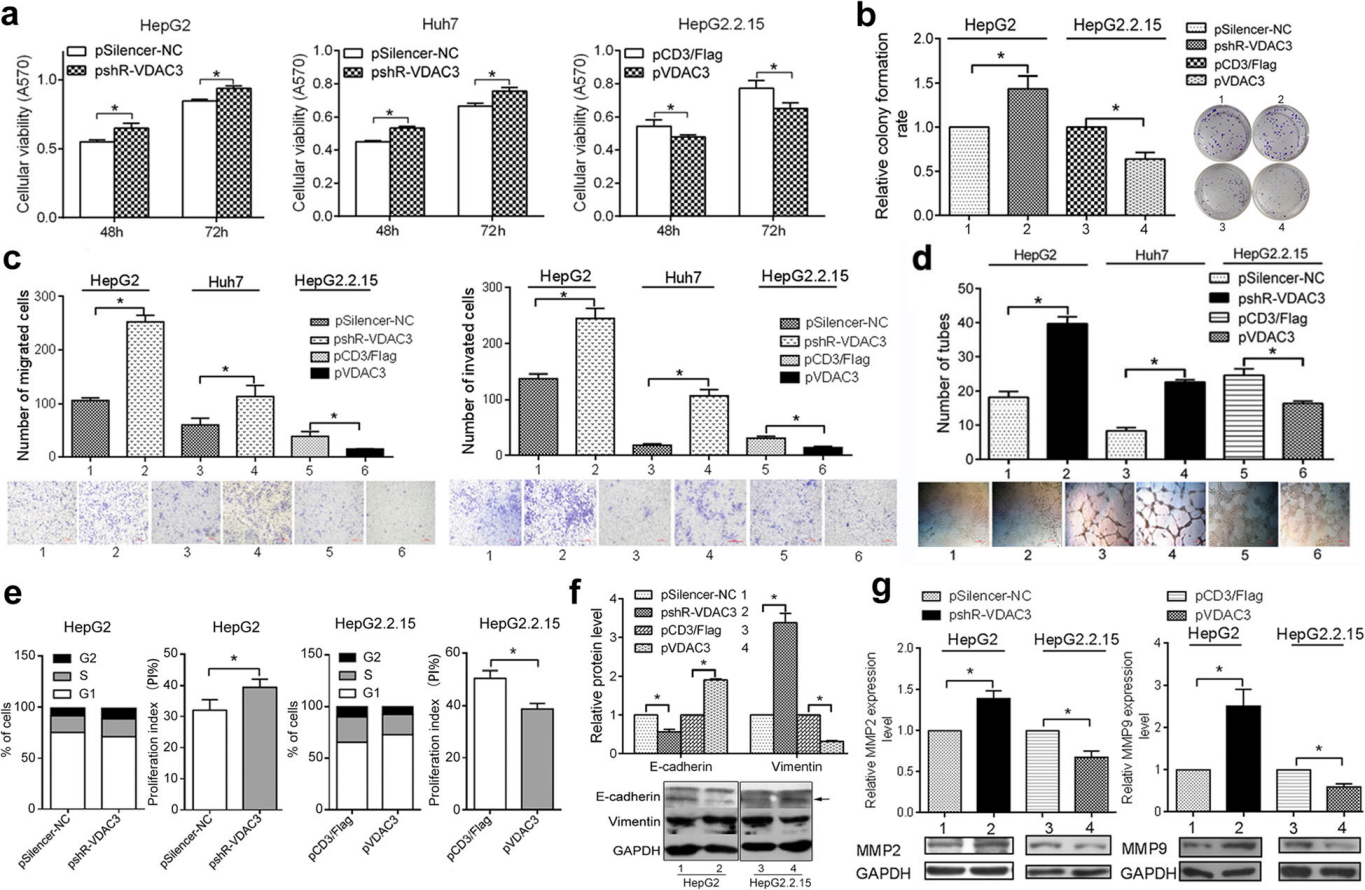
VDAC3 acts as a tumor suppressor. Huh7 and HepG2 cells were transfected with pshR-VDAC3 or controls. HepG2.2.15 cells were transfected with pVDAC3 or controls. Twenty-four hours after transfection, cells were seeded into cell culture inserts. **a** MTT assays were performed to evaluate cell viability. **b** Colony formation assays were used to estimate cell proliferation. **c** Migration/invasion assays and **f** Western blot analyses were used to evaluate EMT. **d** VM and **g** molecular markers of VM were examined to assess tumor angiogenesis. **e** FACS assays were used to examine changes in cell cycle distribution. (*: *P* < 0.05)

**Fig. 5:**
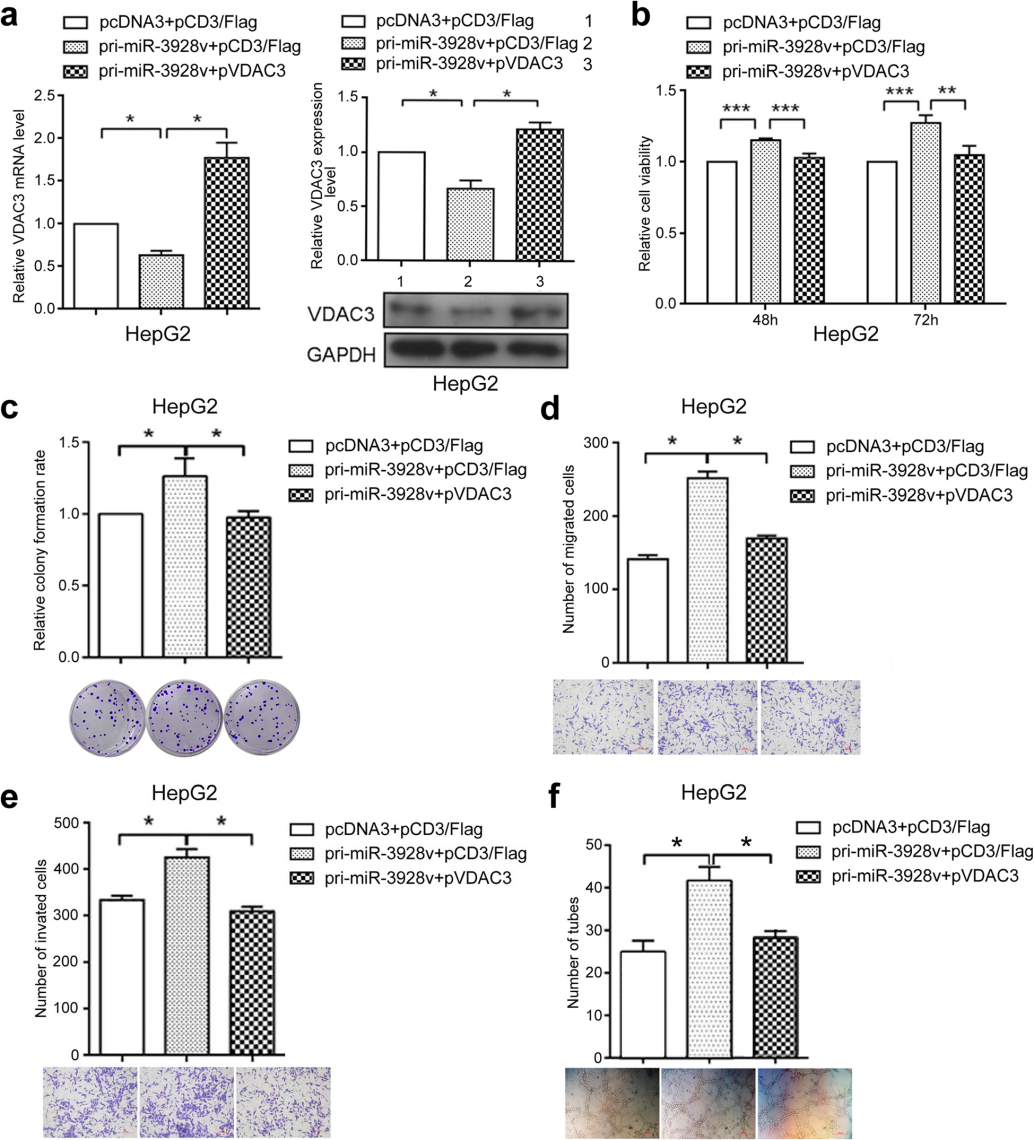
VDAC3 is a functional target of miR-3928v. HepG2 cells were co-transfected with pCD3/Flag or pVDAC3 and pri-miR-3928v. RT-qPCR and Western blot analyses (**a**) and MTT (**b**), colony formation (**c**), migration (**d**), invasion (**e**) and tube formation assays (**f**) were performed to analyze whether VDAC3 could functionally rescue the miR-3928v-induced phenotype. (*: *P* < 0.05, **: *P* < 0.01, ***: *P* < 0.001)

**Fig. 6:**
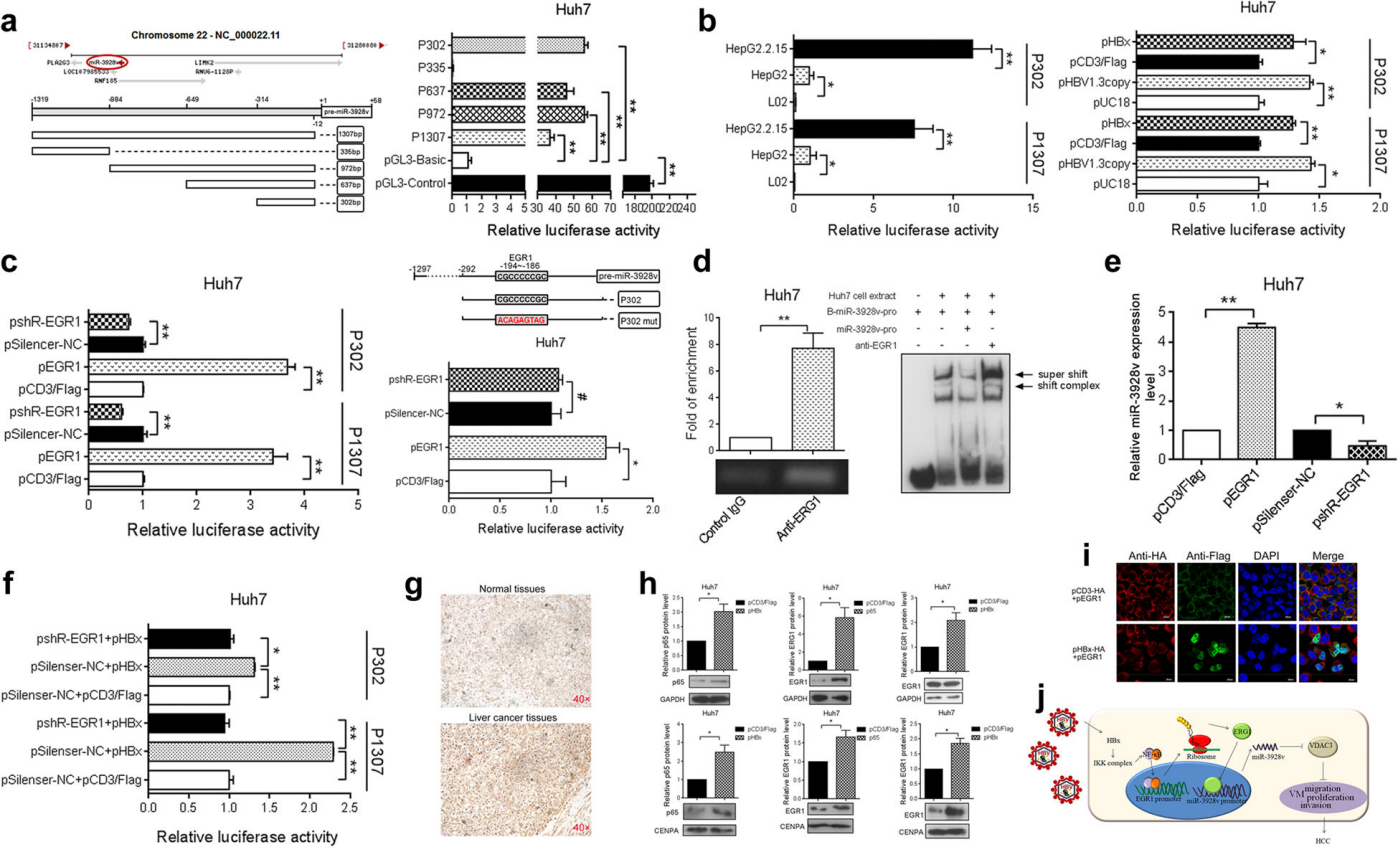
HBx up-regulates miR-3928v expression via EGR1. **a** Schematic diagram of the location of miR-3928v in the human genome; the relative positions of the miR-3928v promoter constructs are shown (left). Luciferase reporter assays were used to examine miR-3928v promoter activity in Huh7 cells (right). **b** Promoter activity in HBV (+) and HBV (−) cells (left). Then, the promoter activity in Huh7 cells was examined after HBV or HBx overexpression (right). **c** The effect of EGR1 on promoter activity (left). The EGR1 binding site in the miR-3928v promoter was mutated, and the luciferase activity induced by EGR1 was examined (right). **d** ChIP analysis (left) and EMSAs (right) revealed that EGR1 directly binds to the miR-3928v promoter. **e** The miR-3928v expression level was measured by RT-qPCR after EGR1 overexpression or knockdown. **f** Blocking EGR1 inhibited HBx-induced miR-3928v promoter activity. **g** Representative IHC images showing the magnitude of EGR1 expression in tissues isolated from liver cancer patients. **h** Total and nuclear expression of EGR1 and P65 in Huh7 cells after transfection with HBx was analyzed by Western blot. **i** An immunofluorescence assay was used to detect the induction of EGR1 expression in the nucleus by HBx in Huh7 cells. **j** Mechanism by which HBx-induced miR-3928v expression contributes to HCC malignancy. (*: *P* < 0.05, **: *P* < 0.01)

## References

[1] Zhang (2018). miR-3928v is induced by HBx via NF-κB/EGR1 and contributes to hepatocellular carcinoma malignancy by down-regulating VDAC3. J Exp Clin Cancer Res.

